# Neutrophil CD64 index as a good biomarker for early diagnosis of bacterial infection in pregnant women during the flu season

**DOI:** 10.1111/irv.13191

**Published:** 2023-08-25

**Authors:** Lifei Yu, Panpan Cen, Linjian Zhang, Jianfei Ke, Xiangfei Xu, Jiexia Ding, Jie Jin, Jianhang Leng, Yunsong Yu

**Affiliations:** ^1^ Department of Infectious Diseases, Affiliated Hangzhou First People's Hospital Zhejiang University School of Medicine Hangzhou China; ^2^ Key Laboratory of Microbial Technology and Bioinformatics of Zhejiang Province Hangzhou China; ^3^ Department of Clinical Laboratory, Affiliated Hangzhou First People's Hospital Zhejiang University School of Medicine Hangzhou China; ^4^ Department of Central Laboratory, Affiliated Hangzhou First People's Hospital Zhejiang University School of Medicine Hangzhou China; ^5^ Department of Infectious Diseases, Sir Run Run Shaw Hospital Zhejiang University School of Medicine Hangzhou China

**Keywords:** bacterial infection, biomarker, CD64, influenza, pregnant women

## Abstract

**Background:**

Pregnant women are at high risk of developing febrile illness during the flu season. Early identification of a viral or bacterial infection is crucial in the management of febrile pregnant patients. Neutrophil CD64 (nCD64) has been shown to have more important diagnostic value in sepsis than traditional inflammatory indicators.

**Methods:**

The pregnant women enrolled were divided into three groups according to disease: influenza A infection, bacterial infection and healthy controls. Peripheral blood CD64, leukocyte, C‐reactive protein (CRP), procalcitonin (PCT) and human Th1/Th2‐related cytokines levels were routinely measured. The correlation between and diagnostic value of the nCD64 index and other biomarkers were evaluated using Spearman's correlation test and receiver operating characteristic (ROC) curve analysis.

**Results:**

Pregnant women with bacterial infection had significantly elevated levels of leukocytes (8.4 vs. 5.95, 10^9^/L; *P* = 0.004), CRP (89.70 vs. 50.05 mg/mL; *P* = 0.031), PCT (0.13 vs. 0.04 ng/mL; *P* = 0.010) and TNF‐α (0.46 vs. 0.38 pg/mL; *P* = 0.012) and an elevated nCD64 index (12.16 vs. 0.81; *P* < 0.001) compared with those with influenza A infection. The area under the receiver operating characteristic (AUROC) curve of the nCD64 index to discriminate bacterial infection among pregnant women (AUROC = 0.9183, *P* < 0.0001) was the largest. The sensitivity and specificity of the nCD64 index at an optimal cut‐off value of 3.16 were 84% and 100%, respectively, with a negative predictive value (NPV) of 94%.

**Conclusions:**

Our study demonstrates the clinical value of the nCD64 index in distinguishing between bacterial infection and influenza A in pregnant women.

**Abbreviations**
ALB: albumin
ALT: alanine transaminase
AUROC: area under the receiver operating characteristic
BMI: body mass index
CI: confidence interval
CRP: C‐reactive protein
Hgb: haemoglobin
HLH: hemophagocytic lymphohistiocytosis
IFN‐γ: interferon‐γ
IL‐10: interleukin‐10
IL‐2: interleukin‐2
IL‐4: interleukin‐4
IL‐6: interleukin‐6
IQRs: interquartile ranges
Lym: lymphocytes
MFI: mean fluorescence intensity
Mo: monocytes
nCD64: neutrophil CD64
NPV: negative predictive value
PCT: procalcitonin
Plt: platelet
PMN: polymorphonuclear neutrophils
ROC: receiver operating characteristic
Scr: serum creatinine
TNF‐α: tumour necrosis factor‐α

## 1 INTRODUCTION

Pregnant women are a special group of people in terms of febrile diseases during the influenza season. Once infected with the influenza virus or bacteria, they have more than four times the hospitalization rates of the general population and are at increased risk of complications.^1^, ^2^ Pathogenic microbial infection is associated with adverse pregnancy outcomes. For example, pregnant women with influenza A accounted for 13% of all deaths during the 2009 US flu season, and *Listeria monocytogenes* infection caused 35% of foetal deaths in Israel.^1^, ^2^, ^3^ Thus, early monitoring of inflammatory biomarkers is essential for febrile pregnant women.

However, traditional biomarkers of inflammation, such as C‐reactive protein (CRP) level and leukocyte count, may make it difficult to identify the causes of inflammation.^4^ Procalcitonin (PCT) is a diagnostic marker for sepsis in critically ill patients,^5^ but is not effective for some local infections. In assessing bacterial coinfection with influenza, PCT was superior to CRP, and PCT < 0.29 ng/mL had a high negative predictive value (NPV) of 94% for excluding coinfection, especially in patients without shock.^6^ Moreover, it has been reported that the inflammatory changes produced in peripheral blood leukocytes during normal pregnancy resemble those of sepsis due to the activation of the innate immune response.^7^ Hence, identifying novel inflammatory biomarkers for the rapid distinction between bacterial infection and influenza in pregnant patients during the influenza season is crucial.

CD64 (FcγRI) is the FcγR family member with the highest affinity for immunoglobulin G and is expressed on monocyte (Mo)‐macrophages, neutrophils and eosinophils.^8^, ^9^ The FcγRI‐antibody‐pathogen complex is formed when the pathogenic organism is infected and mediates a series of downstream inflammatory immune responses. Earlier studies have demonstrated the important diagnostic value of CD64 in the systemic inflammatory response. The CD64 index may play a supporting role in the diagnosis and treatment of neonatal sepsis and is considered to be a more reliable indicator for early diagnosis than leukocyte, CRP and PCT levels.^10^, ^11^ A meta‐analysis of 14 studies of CD64 in septic patients concluded that neutrophil CD64 (nCD64) was an excellent biomarker for identifying sepsis in adult patients, with better accuracy than CRP and PCT levels.^12^ In addition, nCD64 has been reported to be higher in patients with influenza A infection, both with and without pneumonia, than in healthy people.^13^, ^14^ A further study revealed that nCD64 could help differentiate between bacterial and viral infections.^15^

The pregnant state is a delicate immunological balance in which maternal cells must prevent antigenic rejection of the foreign foetus while simultaneously maintaining adequate maternal defence functions to fight against infections.^9^, ^16^ A progressive upregulation of CD64 was detected in the peripheral circulation during pregnancy, supporting the idea that pregnancy leads to increased innate immunity.^17^ Pregnant women with acute bacterial infection had higher levels of CD64 on the surface of neutrophils and Mos than women who were not pregnant.^18^ However, little is known about the ability of the nCD64 index to discriminate between bacterial and viral infections in pregnant women.

In this study, we aimed to evaluate the practical value of the nCD64 index in distinguishing between bacterial infection and influenza A in pregnant women infected early in the influenza season.

## 2 PATIENTS AND METHODS

### 2.1 Study subjects

A total of 96 pregnant women and 30 healthy nonpregnant women were admitted to the Department of Infectious Diseases at Hangzhou First People's Hospital, Zhejiang University School of Medicine, China between September 2018 and March 2019. The pregnant women were divided into three groups according to disease: influenza A infection (*n* = 24), bacterial infection (*n* = 30) and healthy controls (HCs) (*n* = 42). The diagnosis of seasonal influenza A was made according to the criteria defined by the Infectious Diseases Society of America.^1^ Influenza A infection was not concomitant with bacterial infection. The bacterial infection group included patients with positive blood cultures and patients with negative blood cultures who were clinically diagnosed with bacterial infections.

The exclusion criteria for all subjects were multiple pregnancy, diabetes, immune‐related diseases, viral hepatitis, HIV, syphilis and other infectious diseases. The fever course of infectious subjects was 1–5 days. The study subjects underwent routine blood tests, including CRP, PCT, CD64, cytokines, blood culture and other indicators before using antibiotics or oseltamivir.

All methods were performed in accordance with the relevant guidelines and regulations. After admission, the pregnant women and healthy nonpregnant women were notified of all test results and the indicators of infections and provided written informed consent before blood collection. The study was approved by the Ethics Committee of Hangzhou First People's Hospital (2017 Research Medical Review No.116‐01). Written informed consent was obtained from all subjects.

**FIGURE 1:**
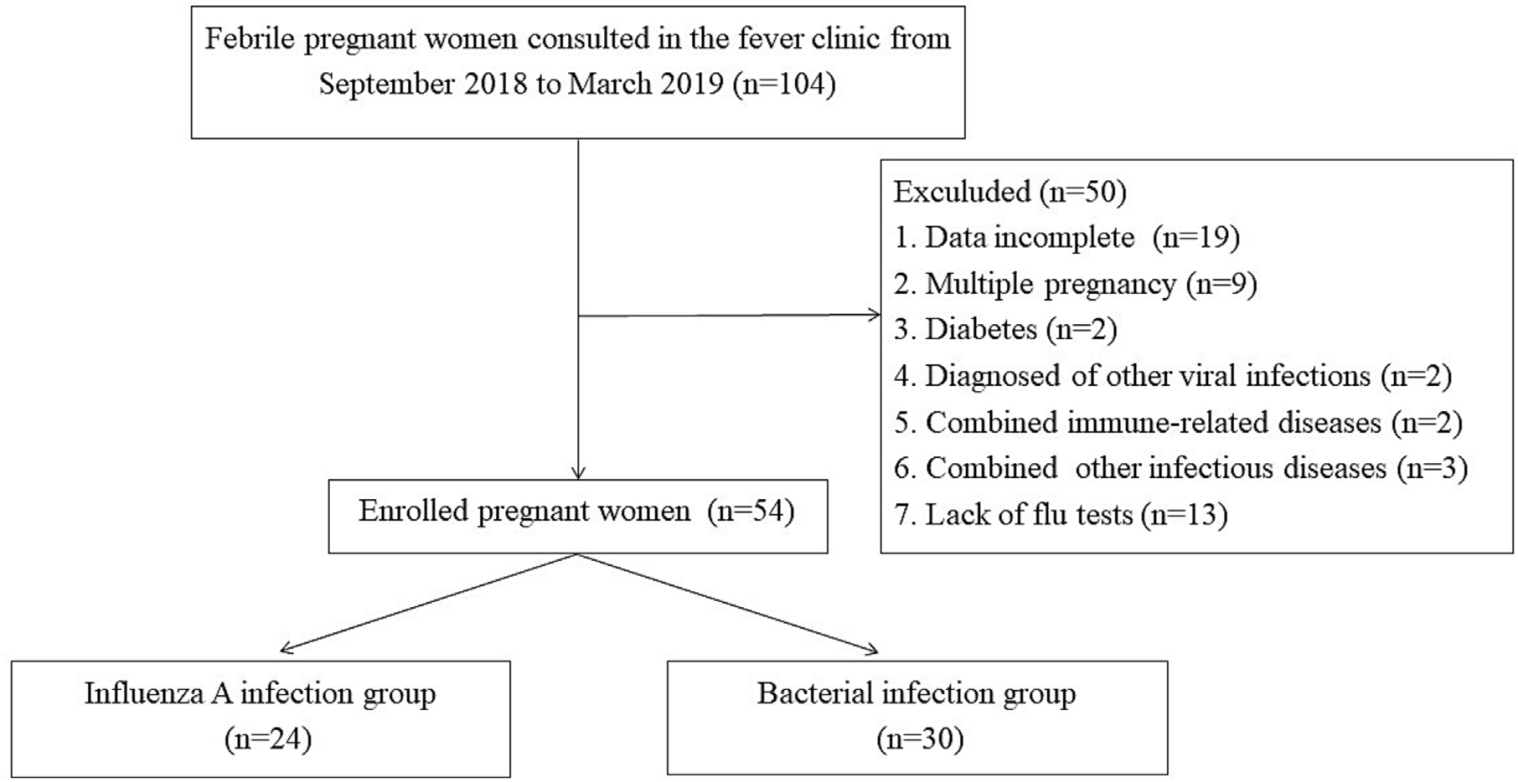
Flow chart for the admission of febrile pregnant women.

The flow chart and QUADAS‐2 of this study are outlined in Figure 1 and Supplementary S1.

### 2.2 Examination of traditional inflammatory indicators: leukocytes, CRP and PCT

The levels of leukocytes, CRP and PCT in the peripheral blood of patients were measured in a clinical laboratory at the Affiliated Hangzhou First People's Hospital, Zhejiang University School of Medicine. Routine blood counts were performed with a Mindray CAL 8000 haematology analyser (Mindray Bio‐Medical Electronics Co., Ltd., Shenzhen, China). CRP was measured by a particle‐enhanced turbidimetric immunoassay. PCT was measured by a chemiluminescent immunoassay using an automatic chemiluminescence apparatus (Caris200) and diagnostic kits (Xiamen Innodx Biotechnology Co., Ltd.). All operations strictly complied with the experimental procedures provided by the manufacturers.

### 2.3 Cytometric bead array to identify and determine human Th1/Th2‐related cytokines

Peripheral blood samples were centrifuged for serum separation. Two millilitres of capture beads were suspended by vigorously vortexing for a few seconds before mixing. Then, a 300 μL aliquot of the capture beads from each assay tube to be analysed was added into a single tube labelled ‘mixed capture beads’. The bead mixtures were incubated with the capture beads in serum enhancement buffer. The mixed capture beads were centrifuged at 200 × g for 5 min. The mixed capture bead pellet was resuspended in serum enhancement buffer. The mixed capture beads were incubated for 30 min at room temperature protected from light, and then 25 μL was added to all assay tubes. The BD™ Cytometric Bead Array Human Th1/Th2 Cytokine Kit II (BD Biosciences, USA) was used to quantitatively measure interleukin‐2 (IL‐2), interleukin‐4 (IL‐4), interleukin‐6 (IL‐6), interleukin‐10 (IL‐10), tumour necrosis factor‐α (TNF‐α) and interferon‐γ (IFN‐γ) protein levels in a single sample. Human Th1/Th2 cytokine standard dilutions (25 μL) were added to the control assay tubes. The samples to be tested were added (25 μL) into the tubes. Next, 25 μL of Human Th1/Th2‐II PE Detection Reagent was added to all assay tubes, and the samples were incubated for 2.5 h at room temperature protected from light. A 1 mL aliquot of wash buffer was added to each assay tube, and the tubes were then centrifuged at 200 *× g* for 5 min. The supernatant from each assay tube was carefully aspirated and discarded. Finally, 100 μL of wash buffer was added to each assay tube to resuspend the bead pellet for testing.

### 2.4 Flow cytometric detection of CD64 MFI and calculation of the nCD64 index

1. Sample preparation: 30 μL anti‐human CD45 percp (CD45‐Percp; BD Biosciences, USA), anti‐human CD14 FITC (CD14‐FITC; BD Biosciences, USA) and anti‐human CD64 phycoerythrin (CD64‐PE; eBioscience, BD Biosciences, USA) were added to the numbered test tubes as needed. Then, 50 μL fresh whole blood with anticoagulant was added to the test tubes. The mixture was incubated at room temperature for 15 min in the dark, and 2 mL erythrocyte lysis buffer (BD Biosciences, USA) was added to lyse red blood cells for 10 min. After centrifugation, the supernatant was discarded to obtain peripheral blood mononuclear cells (PBMCs).
2. Sample detection: PBMCs stained with fluorescent antibodies were examined by flow cytometry (FACSCalibur™, BD Biosciences, USA). The mean fluorescence intensity (MFI) of CD64 on lymphocytes (Lym), Mos and polymorphonuclear neutrophils (PMN) was acquired by flow cytometry analysis software (CellQuest, BD Biosciences, USA). The nCD64 index was obtained according to the formula $\frac{\mathrm{CD}64\text{MFIPMN}/\mathrm{CD}64\text{MFILym}}{\mathrm{CD}64\text{MFIMo}/\mathrm{CD}64\text{MFIPMN}}$.

### 2.5 Statistical analysis

Statistical analysis was performed using SPSS for Windows release 19.0 (SPSS Inc., Chicago, IL, USA) for the data and GraphPad Prism 7 (GraphPad, Inc., CA, USA) for the graphs. Continuous variables are presented as medians with interquartile ranges (IQRs). The comparisons of continuous variables between independent groups were performed using the Mann–Whitney U test (two groups) or Kruskal–Wallis's test (multiple groups) followed by Dunn's post‐test for multiple comparisons. The chi‐square test was used for count data. Spearman's correlation test was used to explore the correlation between the biomarkers. The sensitivity and specificity of the different indicators were compared by area under the receiver operating characteristic (AUROC) curve analysis. Optimal cut‐off values to obtain the highest AUROC were calculated using the Youden index. A *P‐*value <0.05 was considered a statistically significant difference between groups.

## 3 RESULTS

### 3.1 Characteristics of the patients

The baseline characteristics of the 96 enrolled pregnant women and 30 healthy nonpregnant women enrolled as controls are listed in Table 1. The pregnant women were divided into three groups: influenza A (*n* = 24), bacterial infections (*n* = 30) and HCs (*n* = 42). Of the 30 patients with bacterial infections, 25 patients had focal infections (of which 10, five, nine and one had respiratory and urinary tract infections, enteritis and abdominal infection, respectively), and five patients had a bloodstream infection (all with microbiologically confirmed diagnoses).

**TABLE 1:** Baseline characteristics of enrolled patients and controls.

Characteristics	Pregnant women			Controls	*P‐*value^a^
Group	Influenza A (*n* = 24)	Bacterial infection (*n* = 30)	Healthy controls (*n* = 42)	Healthy nonpregnant women (*n* = 30)	NA
Age, years	30(28–33)	27.5(26–31.25)	29(27–34.5)	32(27–35)	0.097
Gestational age, w	24(17.25–33.75)	25.5(19.5–33)	20(10–27)	NA	0.828
Number of births	‐	‐	‐	‐	0.561
Primiparous, n(%)	15(62.5)	21(70)	32(76.2)	NA	‐
Parous, n(%)	9(37.5)	9(30)	10(23.8)	NA	‐
BMI, kg/m^2^	23.79(22.19–25.17)	24.70(23.18–26.22)	NA	NA	0.370
ALT, U/L	20(16–23)	19(11.75–28.00)	NA	NA	0.486
ALB, g/L	2.75(30.55–35.78)	30.05(25.98–34.28)	NA	NA	0.107
Scr, umol/L	59(54.25–64.50)	56.5(53.00–63.25)	NA	NA	0.280
Leukocytes, 10^9/L	5.95(4.75–7.15)	8.40(6.45–10.65)	NA	NA	0.004
Hgb, g/L	111(100.25–118.00)	107(94.75–115.25)	NA	NA	0.185
Plt, 10^9^/L	186(149.50–215.50)	184.5(153.75–251.50)	NA	NA	0.508
CRP, mg/mL	50.05(20.98–84.10)	89.70(44.30–115.05)	NA	NA	0.031
PCT, ng/mL	0.04(0.01–0.12)	0.13(0.05–0.33)	NA	NA	0.010
IL‐6, pg/mL	5.08(3.27–7.65)	7.13(2.76–39.63)	2.92(1.54–3.73)	2.58(1.50–3.78)	0.273
nCD64 index	0.81(0.35–1.83)	12.16(3.70–49.90)	1.64(0.87–3.86)	0.61(0.33–1.38)	<0.001

*Note*: Continuous variables are shown as medians with interquartile ranges (IQRs). Comparisons of continuous variables were performed using the Mann–Whitney U test. The chi‐square test was used for count data.

Abbreviations: ALB, albumin; ALT, alanine transaminase; BMI, body mass index; CRP, C‐reactive protein; Hgb, haemoglobin; IL‐6, interleukin‐6; NA, not available; nCD64, neutrophil CD64; PCT, procalcitonin; Plt, platelet; Scr, serum creatinine.

^a^ Comparison between the influenza A infection group and the bacterial infection group.

There was no significant difference in age or gestational age among the three groups of pregnant women, as shown in Table 1.

### 3.2 Comparison of traditional inflammatory biomarkers between pregnant women with influenza A and with bacterial infection

Pregnant women with bacterial infection had significantly elevated levels of blood leukocytes (*P* < 0.01) and serum levels of CRP (*P* < 0.05) and PCT (*P* < 0.05) compared with those with influenza A infection, as shown in Table 1.

### 3.3 Blood levels of human Th1/Th2‐related cytokines

**FIGURE 2:**
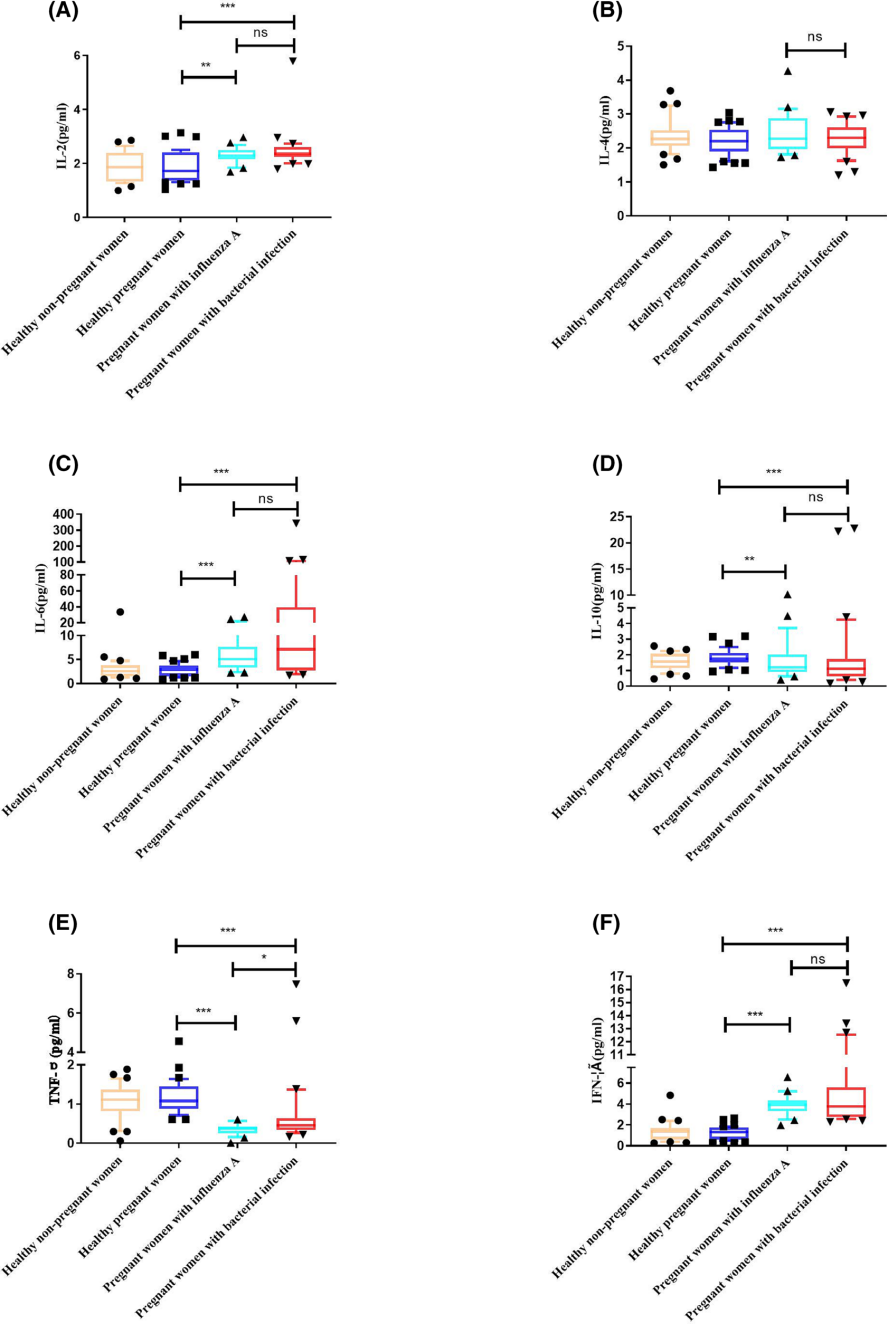
The profiles of human Th1/Th2‐related cytokine expression in blood among 96 enrolled pregnant women and 30 healthy nonpregnant women. The blood levels of interleukin‐2 (IL‐2), interleukin‐4 (IL‐4), interleukin‐6 (IL‐6), interleukin‐10 (IL‐10), tumour necrosis factor‐α (TNF‐α) and interferon‐γ (IFN‐γ) among pregnant women with influenza A, pregnant women with bacterial infection, pregnant healthy controls (HCs) and healthy nonpregnant women are shown. The data are shown as medians with interquartile ranges (IQRs). The comparisons of cytokine expression among four independent groups were performed using the Mann–Whitney U test (two groups) and Kruskal–Walli's test (multiple groups) followed by Dunn's post‐test for multiple comparisons. ns, no significance. *P < 0.05; **P < 0.01; ***P < 0.001.

Pregnant women with bacterial infection had significantly higher serum levels of TNF‐α than those with influenza A infection (0.46 vs. 0.38 pg/mL; *P* = 0.012), but no significant differences were observed in IL‐2, IL‐4, IL‐6, IL‐10 or IFN‐γ (2.36 vs. 2.29, 2.30 vs. 2.28, 7.13 vs. 5.08, 1.12 vs. 1.20 and 3.77 vs. 3.92 pg/mL; *P* > 0.05), as shown in Figure 2.

Compared with healthy pregnant women, pregnant women with bacterial infections had significantly elevated blood levels of IL‐2, IL‐6, IL‐10, TNF‐α and IFN‐γ (2.36 vs. 1.71, 7.13 vs. 2.92, 1.12 vs. 1.74 and 3.77 vs. 1.33 pg/mL; *P* < 0.001, *P* < 0.001, *P* = 0.001 and *P* < 0.001, respectively) but lower levels of TNF‐α (0.46 vs. 1.08 pg/mL, *P* < 0.001) and had no significant difference in IL‐4 (2.30 vs. 2.21 pg/mL; *P* = 0.523); pregnant women with influenza A showed significantly elevated blood levels of IL‐2, IL‐6, IL‐10, TNF‐α and IFN‐γ (2.29 vs. 1.71, 5.08 vs. 2.92, 1.20 vs. 1.74 and 3.92 vs. 1.33 pg/mL; *P* = 0.004, *P* < 0.001, *P* = 0.008, *P* < 0.001 and *P* < 0.001, respectively) but lower levels of TNF‐α (0.38 vs. 1.08 pg/mL; *P* < 0.001) and showed similar levels of IL‐4 (2.28 vs. 2.21 pg/mL; *P* = 0.348).

There was no difference in the blood levels of human Th1/Th2‐related cytokines between healthy pregnant women and healthy nonpregnant women.

### 3.4 Comparison of the nCD64 index between pregnant women with influenza A and with bacterial infection

**FIGURE 3:**
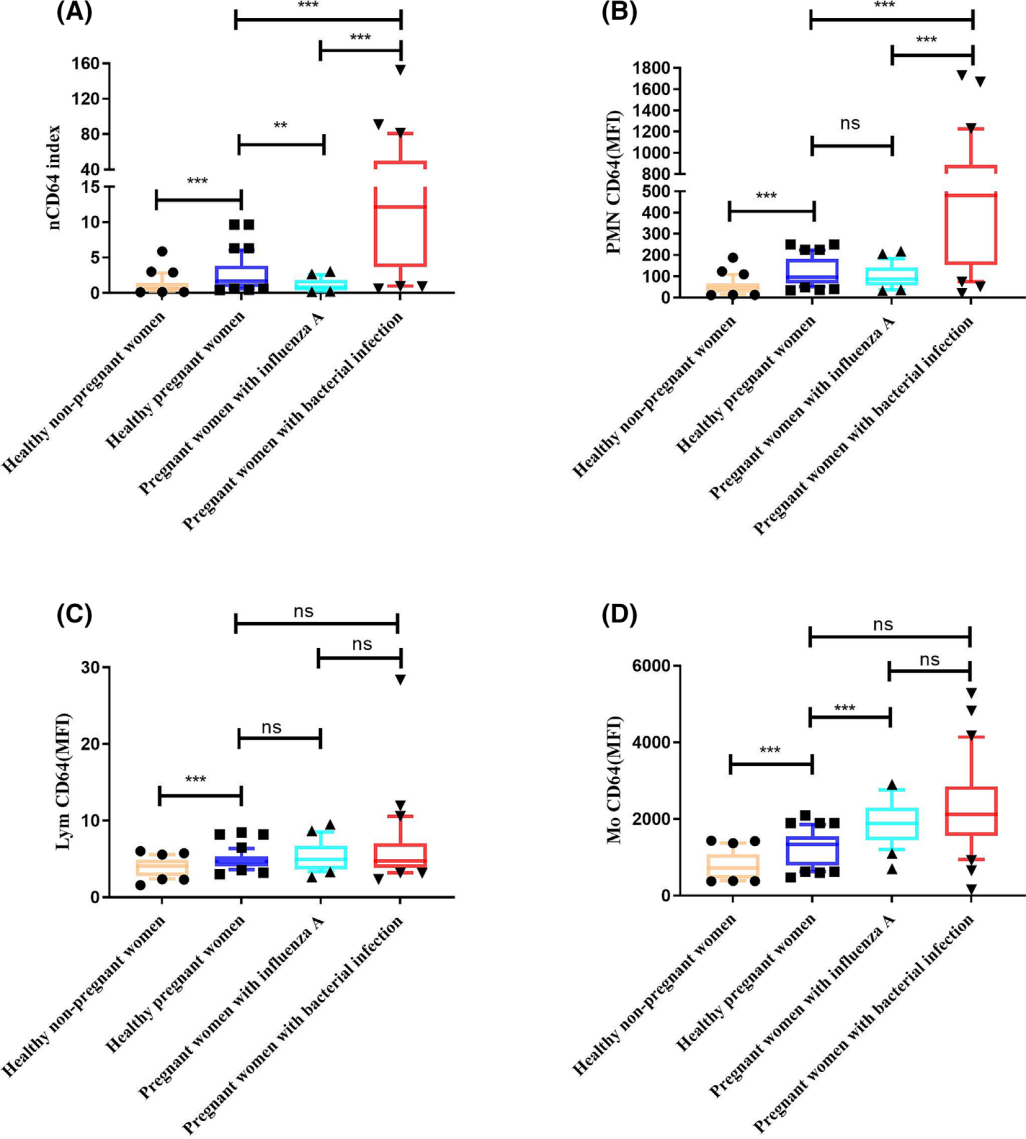
The profiles of CD64 expression among 96 enrolled pregnant women and 30 healthy nonpregnant women. (A) Investigation of the neutrophil CD64 (nCD64) index in pregnant women with influenza A and bacterial infection, pregnant healthy controls (HCs) and healthy nonpregnant women. The different mean fluorescence intensities (MFIs) of (B) PMNCD64, (C) LymCD64 and (D) MoCD64 among pregnant women with influenza A and bacterial infection, pregnant HCs and healthy nonpregnant women. The data are shown as medians with interquartile ranges (IQRs). The comparisons of CD64 expression among four independent groups were performed using the Mann–Whitney U test (two groups) and Kruskal–Wallis test (multiple groups) followed by Dunn's post‐test for multiple comparisons. ns, no significance. *P < 0.05; **P < 0.01; ***P < 0.001.

Pregnant women with bacterial infection had a higher nCD64 index than those with influenza A infection (12.16 vs. 0.81; *P* < 0.001). Compared with healthy pregnant women, pregnant women with bacterial infection had a significantly elevated nCD64 index (12.16 vs. 1.64; *P* < 0.001); pregnant women with influenza A showed a significantly decreased nCD64 index (0.81 vs. 1.64; *P* = 0.003), and healthy nonpregnant women had a significantly lower nCD64 index (0.61 vs. 1.64; *P* < 0.001), as shown in Figure 3.

Pregnant women with bacterial infection had higher flow cytometric detection of PMNCD64 MFI than those with influenza A infection (480.57 vs. 85.86; *P* < 0.001). There was no significant difference in LymCD64 and MoCD64 MFI between the two groups (4.68 vs. 4.88 and 2119.22 vs. 1881.11; *P* = 0.826 and *P* = 0.139, respectively).

Compared with healthy pregnant women, pregnant women with bacterial infection had significantly elevated values of PMNCD64 and MoCD64 MFI (480.57 vs. 94.75 and 2119.22 vs. 1334.01; *P* < 0.001) and no significant difference in LymCD64 MFI (4.68 vs. 4.64; *P* = 0.58); pregnant women with influenza A had significantly elevated values of MoCD64 MFI (1881.11 vs. 1334.01; *P* < 0.001) and no significant difference in PMNCD64 or LymCD64 MFI (85.86 vs. 94.75 and 4.88 vs. 4.64; *P* = 0.413 and *P* = 0.636, respectively). Interestingly, healthy nonpregnant women had significantly lower values of PMNCD64, LymCD64 and MoCD64 MFI (45.33 vs. 94.75, 4.04 vs. 4.64, 713.38 vs. 1334.01; *P* < 0.001).

### 3.5 Correlation analysis between human Th1/Th2‐related cytokines and the nCD64 index in pregnant women with bacterial infection

In the group of 30 pregnant women with bacterial infection, the levels of all human Th1/Th2‐related cytokines (IL‐2, IL‐4, IL‐6, IL‐10, TNF‐α and IFN‐γ) had no significant causal correlation with the nCD64 index (r = −0.0518, *P* = 0.7858; r = −0.0473, *P* = 0.8041; r = 0.1997, *P* = 0.29; r = −0.0888, *P* = 0.6409; r = 0.1636, *P* = 0.3876 and r = 0.1472, *P* = 0.4377, respectively).

### 3.6 AUROC of inflammatory biomarkers in pregnant women with infections (bacterial infection and influenza A)

To compare the value of different inflammatory biomarkers for identifying pregnant women with early infection during the influenza season, 30 pregnant women with bacterial infection and 24 pregnant women with influenza A were included for the AUROC of leukocyte, CRP, PCT, IL‐6 and nCD64 index levels.

**FIGURE 4:**
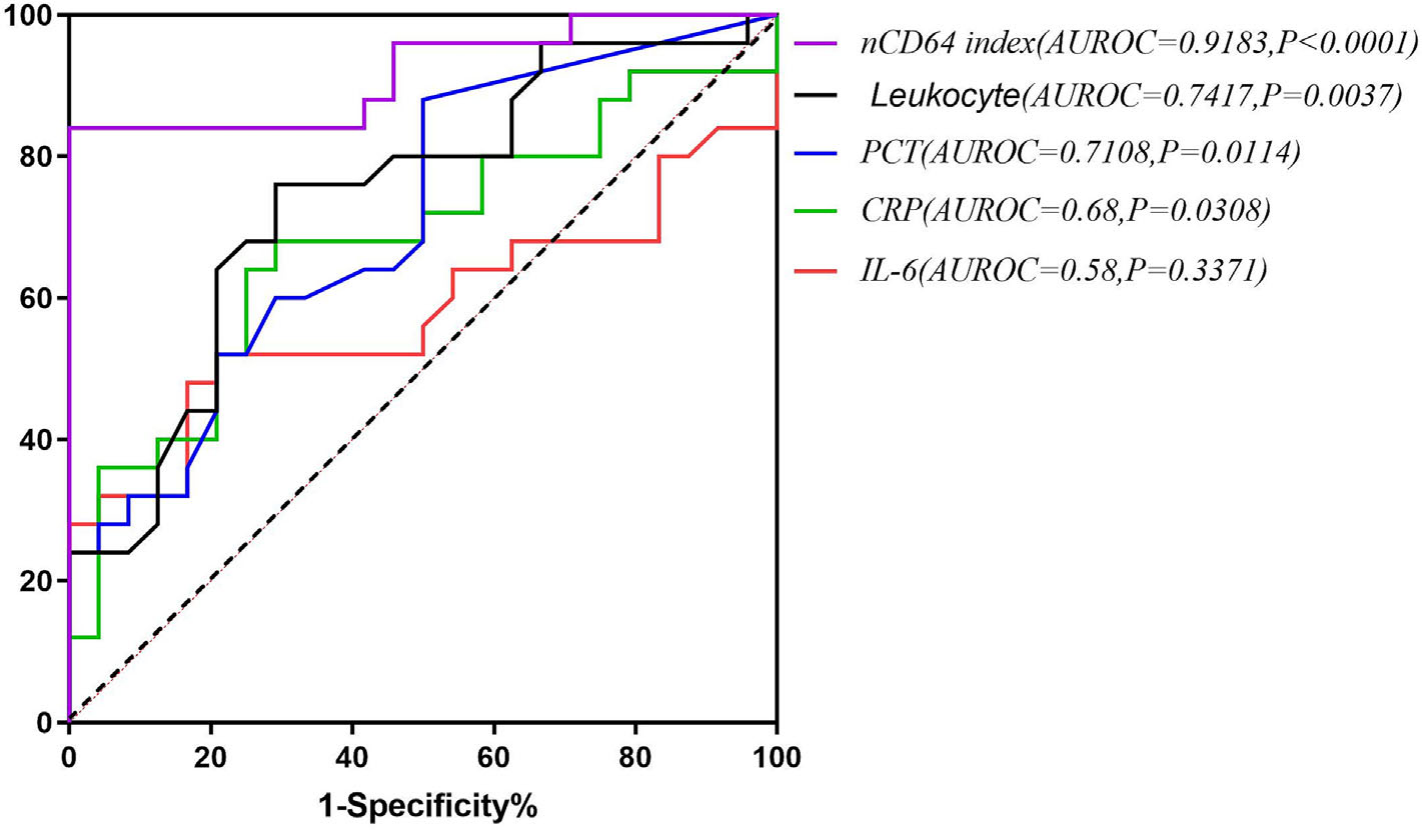
The area under the receiver operating characteristic (AUROC) of leukocyte, C‐reactive protein (CRP), procalcitonin (PCT), interleukin‐6 (IL‐6) and neutrophil CD64 (nCD64) index levels in the infection group. The AUROC of leukocyte, CRP, PCT, IL‐6 and nCD64 index levels are represented by different lines.

We found that the AUROC of the nCD64 index for discriminative ability in bacterial pregnant women (AUROC = 0.9183, *P* < 0.0001, 95% confidence interval (CI) 0.8364 to 1.000) was the largest and was significantly higher than that of leukocytes (AUROC = 0.7417, *P* = 0.0037, 95% CI 0.6015 to 0.8818), PCT (AUROC = 0.7108, *P* = 0.0114, 95% CI 0.5666 to 0.8550) and CRP (AUROC = 0.68, *P* = 0.0308, 95% CI 0.5270 to 0.8330); the AUROC of IL‐6 was the smallest (AUROC = 0.58, *P* = 0.3371, 95% CI 0.4121 to 0.7479) and showed no statistical significance (Figure 4). For pregnant women with early bacterial infection during the influenza season, the sensitivity and specificity of the nCD64 index at an optimal cut‐off value of 3.16 were 84% and 100%, respectively, with an NPV of 94%.

## 4 DISCUSSION

Pregnant women are at high risk of contracting influenza or bacterial infection during the flu season. Once infection occurs, it is easy for pregnant women to trigger a systemic inflammatory response.^1^, ^3^, ^19^, ^20^ The use of peripheral blood biomarkers for early diagnosis of infection in pregnant women is essential. Pregnant women are a unique population and are less studied in this area. Previous research revealed that PCT levels were similar in pregnant and nonpregnant women, and the upper reference limit for PCT was 0.05 ng/mL for any observed gestational age group.^21^ A multicentre prospective observational study showed that a cut‐off value of serum PCT > 0.25 ng/mL had a sensitivity of 87% and a specificity of 79% and was a better biomarker than CRP and leukocytes in differentiating acute pyelonephritis from asymptomatic bacteriuria and acute cystitis in pregnancy.^22^ However, a recent study demonstrated that maternal serum CRP was a better predictive biomarker of histological chorioamnionitis than PCT.^23^

In our study, the three traditional biomarkers (leukocytes, CRP and PCT) in the bacterial infection group were significantly higher than those in the influenza A group. Using receiver operating characteristic (ROC) curves, we found that leukocytes were a better biomarker for the identification of pregnant women with bacterial infection than PCT and CRP. These conflicting results may be attributed to the following reasons: in our study, only five out of 30 pregnant women with bacterial infection had a bloodstream infection; PCT was generally elevated in bloodstream infections, and pregnant women infected with influenza A often experienced a decrease in the leukocyte count, whereas the leukocyte count often increased in women with bacterial infection. In addition, the majority of febrile pregnant women came to the hospital within 1–2 days, and CRP was elevated in the late stage (more than 24 h) of infection compared with other inflammatory markers.^11^, ^24^

It has been reported that pregnant women infected with H1N1 influenza had significantly higher serum levels of IL‐6, IL‐10 and TNF‐α than healthy pregnant women, and IL‐6 was correlated with the severity of the disease.^25^, ^26^ In our study, compared with healthy pregnant women, we observed a significant increase in IL‐2, IL‐6, IL‐10 and IFN‐γ in pregnant women with both influenza A and bacterial infection but lower levels of TNF‐α in both groups. The blood level of TNF‐α in pregnant women with bacterial infection was remarkably elevated compared with those with influenza A infection, but no significant difference was observed for IL‐6, IL‐10 or IFN‐γ. It is easy to understand why most of the cytokines were elevated in infected pregnant women. Strangely, TNF‐α, as an endogenous pyrogen, was lower in healthy pregnant women. The reasons might be that the majority of pregnant women with infections enrolled in our study had relatively mild disease, and TNF‐α is an essential trigger of the cytokine storm. Furthermore, elevated TNF‐α is often associated with adverse pregnancy outcomes,^27^ and in our study, almost all pregnant women had good deliveries at the follow‐up visit, which may be related to maintaining the dynamic balance of TNF‐α/IL‐10. Many prospective studies have shown that the role of cytokines in the distinction between viral and bacterial pathogens is limited, including IL‐6 and IFN‐γ.^28^ We reported similar findings in our study.

As an important Fc receptor mediating the inflammatory immune response, CD64 showed a biphasic expression pattern in peripheral blood, with an initial increase after 2 h, a more pronounced increase after 6 h and a decline within 48 h of stimulus removal.^29^, ^30^ In comparison with traditional biomarkers of inflammation, the nCD64 index has been found to be a relatively reliable biomarker of sepsis,^11^, ^12^ although reports on infection in pregnant women are scarce. A previous study suggested that the nCD64 index may not be a reliable indicator for distinguishing bacterial from viral infections.^4^ However, there were no pregnant women involved in that study; almost half of the patients in the viral infection group were infected with Epstein–Barr virus and had chronic infection, and approximately 1/5 were associated with hemophagocytic lymphohistiocytosis (HLH). The author speculated that it may cause a severe cytokine storm that strongly induces the expression of nCD64. Thus, the nCD64 index of patients with a viral infection was high and similar to that of patients with a bacterial infection. However, in our study, the causal relationship analysis showed no correlation between proinflammatory cytokines and nCD64 in pregnant women with bacterial infection, indicating that nCD64 is an independent biomarker. We found that pregnant women with bacterial infection had a significantly higher nCD64 index than those with influenza A infection and healthy pregnant women, which suggests that the nCD64 index is valuable in the identification of pregnant women. Further analysis of the AUROC of inflammatory biomarkers in pregnant women with infections showed that the nCD64 index had the largest AUROC to discriminate bacterial infection among pregnant women (AUROC = 0.9183, *P* < 0.0001) and was significantly higher than that of leukocytes, PCT and CRP, whereas the AUROC of IL‐6 was the smallest and showed no statistical significance. This provided the basis for the conclusion that nCD64 could be an optimal blood biomarker for the early diagnosis of bacterial infection in pregnant women during the influenza season. The sensitivity and specificity of the nCD64 index at an optimal cut‐off value of 3.16 were 84% and 100%, respectively, with an NPV of 94%. There are seldom researches on the application of the nCD64 index among the pregnant women with viral infections. We consider this test would be valid in other type of viral flu among the pregnant population. This is an area we need to validate further in the future.

There were several limitations to the study we conducted. First, this was a single‐centre study, which could lead to selection bias. Second, the number of pregnant women enrolled was small, which might introduce a certain degree of statistical bias. Third, cases of influenza with bacterial infection were not included, making the value of the nCD64 index in this population unclear.

In summary, the differences in traditional indicators of inflammation (leukocytes, CRP and PCT), cytokines and the nCD64 index in the peripheral blood of pregnant women with bacterial infection or influenza A during the influenza season were investigated. These findings suggest that the nCD64 index could be an excellent biomarker for the early diagnosis of bacterial infection in pregnant women that is more reliable than leukocytes, CRP, PCT and IL‐6. In the long term, we hope to conduct a multicentre study and include cases of influenza coinfection with bacteria to better understand the value of the nCD64 index in pregnant women with infections.

## AUTHOR CONTRIBUTIONS

Lifei Yu conceived and designed the research. Material preparation, data collection and analysis were performed by Linjian Zhang, Jianfei Ke, Xiangfei Xu and Jiexia Ding. The first draft of the manuscript was written by Lifei Yu and Panpan Cen. All authors commented on previous versions of the manuscript. Jie Jin provided financial support. Yunsong Yu and Jianhang Leng critically revised the work and gave final approval of the manuscript.

## CONFLICT OF INTEREST STATEMENT

No competing financial interests exist.

### PEER REVIEW

The peer review history for this article is available at https://www.webofscience.com/api/gateway/wos/peer-review/10.1111/irv.13191.

## ETHICS APPROVAL STATEMENT

The study was approved by the Ethics Committee of Hangzhou First People's Hospital (2017 Research Medical Review No.116‐01). Informed consent was obtained from all individual participants included in the study.

## Supporting information


**Data S1.** Supplementary Information.

## Data Availability

The datasets used and/or analysed during the present study are available from the corresponding author upon reasonable request.
