# Supplementary material for: Neutrophil CD64 index as a good biomarker for early diagnosis of bacterial infection in pregnant women during the flu season
Source: Influenza Other Respir Viruses. 2023 Aug 25;17(8):e13191. doi: 10.1111/irv.13191 (PMC10457501; doi:10.1111/irv.13191)
Supplement: Supplementary file 1 — Data S1. Supplementary Information. [file IRV-17-e13191-s001.docx]

QUADAS-2

# Phase 1: State the review question:

| *Patients (setting, intended use of index test, presentation, prior testing):* pregnant women with bacterial infection and influenza A (we aimed to evaluate the practical value of the nCD64 index is better than traditional biomarkers of inflammation, such as leukocytes, CRP, PCT, IL-6, in distinguishing between bacterial infection and influenza A in pregnant women infected early in the influenza season.) |
| --- |
| *Index test(s):* nCD64 index |
| *Reference standard and target condition:*1. leukocytes, CRP, PCT, IL-6. 2. nCD64 index could be an excellent biomarker for the early diagnosis of bacterial infection in pregnant women that is more reliable than leukocytes, CRP, PCT and IL-6. |

**Phase 2: Draw a flow diagram for the primary study**


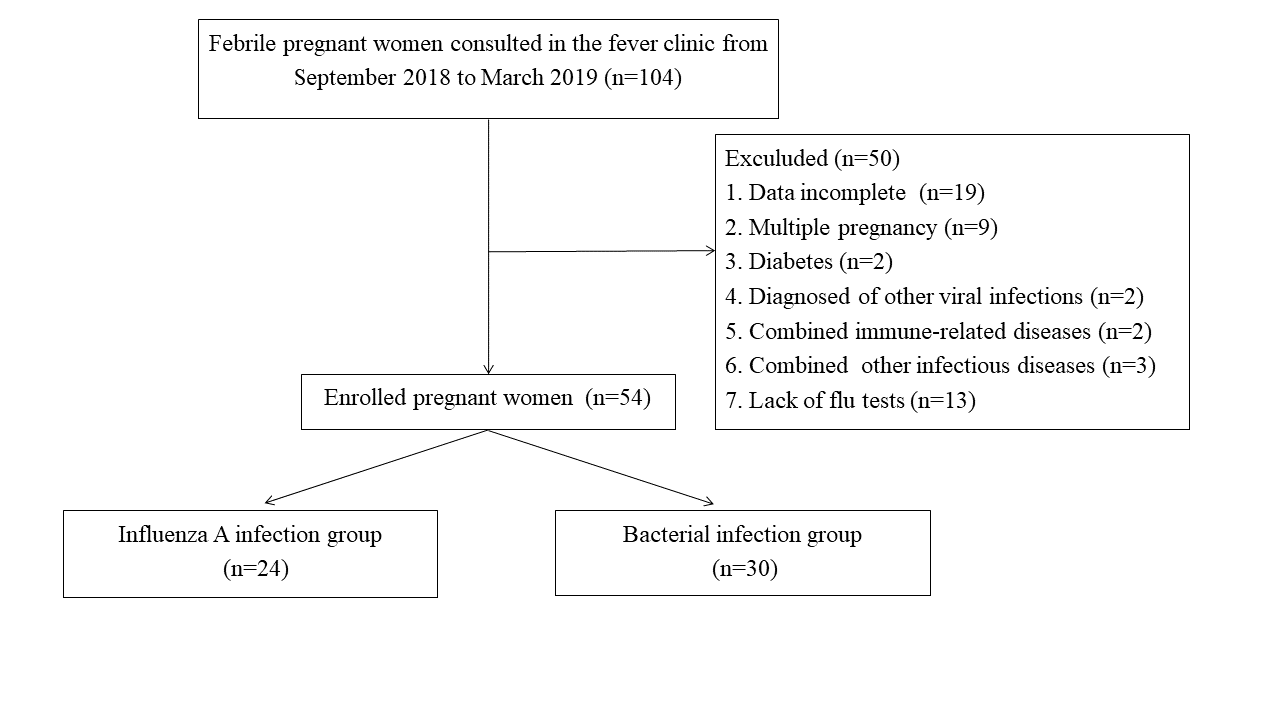


# Phase 3: Risk of bias and applicability judgments

QUADAS-2 is structured so that 4 key domains are each rated in terms of the risk of bias and the concern regarding applicability to the research question (as defined above). Each key domain has a set of signalling questions to help reach the judgments regarding bias and applicability.

| **DOMAIN 1: PATIENT SELECTION**  **A. Risk of Bias** |  |  |
| --- | --- | --- |
| Describe methods of patient selection:  See the flow diagram for the enrolment of febrile pregnant women |  |  |
| - Was a consecutive or random sample of patients enrolled? | | √Yes/No/Unclear |
| - Was a case-control design avoided? |  | √Yes/No/Unclear |
| - Did the study avoid inappropriate exclusions? |  | √Yes/No/Unclear |
| **Could the selection of patients have introduced bias?** | **RISK: √LOW/HIGH/UNCLEAR** | |
| **B. Concerns regarding applicability** |  |  |
| Describe included patients (prior testing, presentation, intended use of index test and setting)**:**  The diagnosis of seasonal influenza A was made according to the criteria defined by the Infectious Diseases Society of America. Influenza A infection group was not concomitant with bacterial infection. The bacterial infection group included patients with positive blood cultures and patients with negative blood cultures who were clinically diagnosed with bacterial infections. | | |
| **Is there concern that the included patients do not match the review question?** | **CONCERN: √LOW/HIGH/UNCLEAR** | |

**DOMAIN 2: INDEX TEST(S)**

**If more than one index test was used, please complete for each test.**

|  | **A. Risk of Bias** |  |  |
| --- | --- | --- | --- |
| Describe the index test and how it was conducted and interpreted:  PBMCs stained with fluorescent antibodies were examined by flow cytometry (FACSCalibur^TM^, BD Biosciences, USA). The mean fluorescence intensity (MFI) of CD64 on lymphocytes (Lym), monocytes (Mo) and neutrophils (PMN) was acquired by flow cytometry analysis software (CellQuest, BD Biosciences, USA). The nCD64 index was obtained according to the formula $\frac{CD64MFIPMN/CD64MFILym}{CD64MFIMo/CD64MFIPMN}$.  the AUROC of the nCD64 index for discriminative ability in bacterial pregnant women (AUROC =0.9183, *P*< 0.0001, 95% C.I 0.8364 to 1.000) was the largest (Figure 4). | | |  |
|  | - Were the index test results interpreted without   knowledge of the results of the reference standard? | Yes/√No/Unclear |  |
|  | - If a threshold was used, was it pre-specified? | Yes/√No/Unclear |  |
|  | **Could the conduct or interpretation of the index test**  **have introduced bias?** | **RISK:√ LOW /HIGH/UNCLEAR** |  |
|  | **B. Concerns regarding applicability** |  |  |
|  | **Is there concern that the index test, its conduct, or interpretation differ from the review question?** | **CONCERN:√ LOW /HIGH/UNCLEAR** |  |

| **DOMAIN 3: REFERENCE STANDARD**  **A. Risk of Bias** |  |  |
| --- | --- | --- |
| Describe the reference standard and how it was conducted and interpreted:  Routine blood counts were performed with a Mindray CAL 8000 haematology analyser (Mindray Bio-Medical Electronics Co., Ltd., Shenzhen, China). CRP was measured by a particle-enhanced turbidimetric immunoassay. PCT was measured by a chemiluminescent immunoassay using an automatic chemiluminescence apparatus (Caris200) and diagnostic kits (Xiamen Innodx Biotechnology Co., Ltd.). The BD™ Cytometric Bead Array Human Th1/Th2 Cytokine Kit II (BD Biosciences, USA) was used to quantitatively measure IL-6.  To identifying pregnant women were bacterial infection, leukocytes (AUROC =0.7417, *P*=0.0037, 95% C.I 0.6015 to 0.8818), PCT (AUROC =0.7108, *P*=0.0114, 95% C.I 0.5666 to 0.8550), CRP (AUROC =0.68, *P*=0.0308, 95% C.I 0.5270 to 0.8330); IL-6 was the smallest (AUROC =0.58, *P*=0.3371, 95% C.I 0.4121 to 0.7479) (Figure 4). | | |
| - Is the reference standard likely to correctly classify the target   condition? | | √Yes/No/Unclear |
| - Were the reference standard results interpreted without knowledge of the results of the index test? | | Yes/√No/Unclear |
| **Could the reference standard, its conduct, or its**  **interpretation have introduced bias?** | **RISK:√ LOW /HIGH/UNCLEAR** | |
| **B. Concerns regarding applicability** |  |  |
| **Is there concern that the target condition as defined by the reference standard does not match the review question?** | **CONCERN:√ LOW /HIGH/UNCLEAR** | |

| **DOMAIN 4: FLOW AND TIMING**  **A. Risk of Bias** |  |  |
| --- | --- | --- |
| Describe any patients who did not receive the index test(s) and/or reference standard or who were excluded from the 2x2 table (refer to flow diagram):  The exclusion criteria for all subjects were multiple pregnancy, diabetes, immune-related diseases, viral hepatitis, HIV, syphilis and other infectious diseases.  Describe the time interval and any interventions between index test(s) and reference standard:  The fever course of infectious subjects was 1-5 days. The study subjects underwent routine blood tests, including CRP, PCT, CD64, cytokines, blood culture and other indicators at the same time, before using antibiotics or oseltamivir. | | |
| - Was there an appropriate interval between index test(s)   and reference standard? | | Yes/√No/Unclear |
| - Did all patients receive a reference standard? |  | √Yes/No/Unclear |
| - Did patients receive the same reference standard? |  | √Yes/No/Unclear |
| - Were all patients included in the analysis? |  | √Yes/No/Unclear |
| **Could the patient flow have introduced bias?** | **RISK: √LOW /HIGH/UNCLEAR** | |
